# Therapist competencies in the context of group-based exercise programs in medical rehabilitation: a qualitative study with patients and exercise therapists from Germany

**DOI:** 10.1186/s13102-023-00674-8

**Published:** 2023-04-21

**Authors:** André Arik Schuber, Madeleine Gernert, Andrea Schaller

**Affiliations:** 1 Institute of Movement Therapy and Movement-Oriented Prevention and Rehabilitation, German Sport University Cologne, Am Sportpark Müngersdorf 6, 50933 Cologne, Germany; 2 Institute of Sport Science, Department of Human Sciences, University of the Bundeswehr Munich, Munich, Germany

**Keywords:** Professional competence, Exercise therapy, Musculoskeletal diseases, Rehabilitation, Qualitative research

## Abstract

**Background:**

Group-based exercise programs account for nearly half of exercise therapy services provided in German medical rehabilitation facilities. However, information about necessary therapist competencies for the successful execution of these programs is sparse. Thus, the aim of this qualitative study was to explore relevant therapist competencies in the context of group-based exercise programs from the patients’ and therapists’ perspective.

**Methods:**

Semi-structured interviews were conducted with five rehabilitation patients following a 3-week inpatient orthopedic rehabilitation program as well as five exercise therapists with work experience in group-based exercise therapy. Data were analyzed using structuring content analysis according to Kuckartz.

**Results:**

From 155 topic-related text passages, collected over 10 interviews, four competency categories with 16 subcategories and respective characteristics were identified. In addition to professional expertise like biomedical knowledge, exercise therapists were expected to possess a multitude of didactic-methodological, personal, and social-communicative abilities.

**Conclusion:**

Our results suggest that the psychosocial, behavioral and educational goals of group-based exercise programs necessitate a wide range of therapist competencies. These conform to the multidimensional nature of exercise therapy and should therefore be covered in vocational and continuing education.

## Background

There is strong evidence that regular physical activity and exercise improve functional capacity and reduce disability across a large number of non-communicable diseases [1]. Accordingly, exercise therapy represents a cornerstone of many cardiac medical rehabilitation programs [2] and is a vital component in the treatment of musculoskeletal conditions like chronic low back pain [3] or osteoarthritis [4, 5].

In Germany, exercise therapy accounts for more than a third (35%) of therapeutic services provided in rehabilitation settings and contains the most extensive patient-therapist interactions [6]. Defined as medically indicated and prescribed physical training, exercise therapy in Germany is usually planned and carried out by trained sports scientists or physical therapists, and organized as either an individual or group-based exercise program (GEP) [7]. In order to achieve optimal rehabilitation outcomes and long-term changes of physical activity behavior, these treatments are embedded into biopsychosocial rehabilitation concepts [8], covering physical training as well as psychosocial, behavioral and educational goals [9]. With an average total duration of 5 h per week, GEP in particular account for nearly half (41–46%) of exercise therapy services provided in German inpatient rehabilitation facilities [6]. Common examples of rehabilitation-specific GEP include back schools [10], aquatic therapy [11] or nordic walking [12]. Allowing for the simultaneous treatment of up to twelve patients, these programs can either be organized as open groups, in which new patients may join at any time, or closed groups, with a fixed number of patients and a set duration [10].

Since GEP share similarities with group-based psychotherapy [13], their potential effects and mechanisms have been derived from Yalom’s therapeutic factors [14]. Examples include the instillation of hope regarding the treatment outcome, the promotion of group cohesion, or the patient’s realization that other group members share similar issues and disabilities (universality) [14]. In particular, Hölter proposed a three-level model including functional, relationship and metacognitive effects of GEP [15]. Similar multidimensional models have since been composed by Huber [16] as well as Pfeifer et al. [17], clearly highlighting the biopsychosocial determinants and approach of exercise therapy. Therefore, in order to utilize GEP to achieve the respective rehabilitation goals, exercise therapists presumably need a broad range of group-specific psychosocial competencies [18, 19].

Competence can be defined as the knowledge, skills, abilities and other characteristics that are needed for effective job performance [20]. Although rehabilitation-specific competency frameworks for physical and exercise therapists have been published by the respective national governing bodies [21–24], information about relevant skills needed for the successful planning, execution and evaluation of group-based treatments like GEP is sparse [19]. Furthermore, data on patient perspectives concerning the competencies required of exercise therapists have so far been mostly limited to the 1:1 setting in musculoskeletal physical therapy [25–27].

Consequently, there is a research gap regarding the patients’ perceptions of key therapist competencies in GEP. In addition, the therapist perspective itself has great importance. Thus, the purpose of this qualitative study was to develop an understanding of relevant therapist knowledge, skills, abilities and behaviors needed in the context of GEP. The research question derived from this was:

- What are relevant therapist competencies in the context of GEP from the patients’ and the therapists’ perspective?

## Methods

This qualitative study was conducted as part of the FeedYou project, which is designed to evaluate the effects of multidimensional exercise therapy feedback on subjective treatment outcome and patient satisfaction in medical rehabilitation [28]. The project is registered in the German Clinical Trial Register (DRKS00027263; Date of Registration: 25/03/2022) and was approved by the German Sport University Research Ethics Committee (#179/2021, approval date February 10, 2022). Methods, data-analysis and findings are reported in accordance with the consolidated criteria for reporting qualitative studies (COREQ) [29].

### Sample

To be eligible to participate in this study, patients were required to meet the following criteria: (1) completion of a 3-week inpatient orthopedic rehabilitation program, (2) suffering from chronic back and/or shoulder pain diagnosed with indication-specific pain questionnaires during the program’s medical examination, and (3) full participation in the indication-specific GEP of said program. The GEP consisted of ten course units on consecutive days, each lasting between 30 and 45 min and including educational as well as exercise therapy components with the goal of promoting long-term physical activity adherence. The corresponding eligibility criteria for exercise therapists were: (1) an exercise therapy related qualification (e.g. certified physical therapist, sports scientist, sports- and exercise therapist) and (2) a minimum of 3-year experience in GEP. All participants were recruited in an inpatient orthopedic rehabilitation clinic in North Rhine-Westphalia, Germany. Patients were informed in person about the purpose of the study by the head of therapy services during the mandatory farewell event on the penultimate day of the rehabilitation program. In addition, each patient received a written participant information sheet. Using self-selection sampling, from 98 patients fitting the inclusion criteria, only five patients chose to participate in this study. Patient interviews took place on the evening of the farewell event. All of the clinic’s 31 exercise therapists with experience in GEP were contacted in person by the head of therapy services as their immediate supervisor, and handed out the participant information sheet. Recognizing that certain therapist characteristics can affect opinions and viewpoints about group therapy, the goal was to recruit a diverse sample with different backgrounds and levels of work experience. Participation in the study was voluntary and self-selected. Patients and exercise therapists were recruited until data saturation was reached. Ten semi-structured interviews with rehabilitation patients (n = 5) and exercise therapists (n = 5) were conducted between February and March 2022. The characteristics of all participants are summarized in Table 1.

**Table 1:** Sample description and interview duration

	Patients (n = 5)	Exercise therapists (n = 5)
Age (years) mean (± SD); minimum–maximum	50 (± 14); 35–63	36 (± 15); 26–62
Gender: female (n; %)	4, (80%)	2, (40%)
Pain location (spine; shoulder)	2; 3	Not applicable
Pain duration (years) mean (± SD); minimum–maximum	9 (± 13); 0.5–30	Not applicable
Number of completed orthopedic rehabilitations programs [n] mean (± SD); minimum–maximum	2 (± 1); 1–3	Not applicable
Group exercise therapy work experience (years) mean (± SD); minimum–maximum	Not applicable	11 (± 10); 4–27
Interview duration (minutes) mean (± SD); minimum–maximum	17 (± 7); 10–27	24 (± 5); 18–30

*SD* standard deviation

### Interview guide

Based on exercise therapy standards provided by framework concepts of both medical rehabilitation [9] and movement-related prevention [30], questions to identify essential therapist competencies were derived. A guide for patients and a guide for therapists were developed, respectively. Questions asked by the researcher were open-ended and allowed for follow-up questions and participants to articulate their viewpoints in their own words [31]. Participants had the freedom to explore additional topics as they saw fit. Table 2 shows the main topics and corresponding key questions of the patient and therapist interview guides.

**Table 2:** Main topics and key questions of the interview guides (translated from German)

Main topic	Key question
General role of exercise therapist	What role does the exercise therapist play for you in the context of group-based exercise programs?
Exercise therapist characteristics	What do you see as important exercise therapist characteristics in the context of group-based exercise programs?
Exercise therapist competencies	What do you see as important exercise therapist competencies in the context group-based exercise programs?
Interacting with participants	How do you think the exercise therapist should interact with the group’s participants?
Influencing the participant’s behavior	How can the exercise therapist influence the participants’ behavior in the context of group-based exercise programs?
Motivating participants	How can the exercise therapist motivate participants in the context of group-based exercise programs?

Prior to data collection, the therapist interview guide was tested using two pilot interviews, both of which were supervised by an experienced researcher. The interviewer (AAS.) was trained to adopt a neutral position, allowing the participant to speak freely and contribute as much information as possible. An additional round of two pilot interviews with rehabilitation patients was conducted to ensure comprehensibility of the interview guide and to determine the interview duration.

### Data collection

Interviews took place in the clinic’s physical therapy examination room and were conducted in German by the same interviewer (AAS) to ensure consistency of questioning. Each interview was audio-recorded in its entirety using a digital audio recorder. Although the interviewer was a trained sports scientist with work experience in musculoskeletal rehabilitation, he had not been involved in the rehabilitation of the patients and had no prior connection to the clinic’s therapy staff. Furthermore, the interviewer introduced himself as an external researcher, interested in the participants’ experiences with GEP as part of orthopedic rehabilitation. All participants provided written informed consent. Data collection ended when participants indicated that they had no additional information to contribute and confirmed that they wished to conclude the interview. None of the participants withdrew from the interview or decided to retract their interview recording. Field notes on situational and non-verbal aspects as well as noteworthy participant behavior were taken following each interview. After five interviews in each stakeholder group, no additional new themes emerged from the data, indicating that saturation had been reached, at which point participant recruitment was concluded. Interviews were transcribed verbatim by a professional typist using the system of Dresing and Pehl [32]. Participant anonymity was preserved by using alphanumeric identifiers and omission of identifying details from transcriptions. Only the first author had access to the identifying code list.

### Data analysis

The anonymized transcripts were analyzed using structuring content analysis [33]. The method shares similarities with the framework method to analyze qualitative data and belongs to the research-methodological approach of qualitative content analysis [34]. After familiarization with the data, a sample of two patient transcripts and two therapist transcripts was independently coded by two researchers (AAS and MG), using an initial coding system developed deductively from the main topics and key questions of the interview guides as seen in Table 2. This was followed by data-driven (‘inductive’) development of categories and subcategories through identification of recurring and important themes. The resulting coding system was then discussed and vetted by the research team, and code definitions as well as sample quotes were added to each category and subcategory to ensure rigor. Once consensus was reached, the entire dataset was independently coded by three researchers using the software MAXQDA (Version 2020, VERBI GmbH, Berlin, Germany).

## Results

The final coding system (Table 3) was developed from 155 topic-relevant interview passages and included the four competency categories (a) professional expertise, (b) didactic-methodological competence, (c) personal competence, and (d) social-communicative competence. Each competency category was further divided into subcategories showing different characteristics where applicable. Although the participants’ statements were distributed evenly among the four competency categories, the subcategories theoretical knowledge, exercise selection, teaching method, patient-centered care, and feedback were mentioned most frequently.

**Table 3:** Coding system with competency categories, subcategories and respective characteristics

Competency categories	Subcategories	Characteristics
Professional expertise	Theoretical knowledge	Anatomy, physiology and pathology
		Pathology-specific exercise therapy
	Practical skills	Exercise demonstration
Didactic-methodological competence	Topic selection	Patient participation
		Coherent structure and sequence of topics
		Topic variety
	Exercise selection	
	Teaching method	Exercise instruction
		Dealing with language barriers
	Volitional strategies	Illustrated exercise handouts
		Affordable and readily available training equipment
Personal competence	Empathy	
	Friendliness	
	Humor	
	Affinity for sports and exercise	
	Authenticity	
Social-communicative competence	Motivation	Verbal encouragement
		Monitoring and highlighting progress using objective performance tests
		Group cohesion
	Patient-centered care	Addressing fears and uncertainties
		Taking care of every patient
		Addressing patients by their family name
	Group facilitation	
	Feedback	Giving feedback
		Asking for feedback
	Professional boundaries	

### Professional expertise

The main category *professional expertise* comprised exercise therapy skills and knowledge acquired as part of vocational education and expanded through continuing education and work experience. Two subcategories were identified: *theoretical knowledge* and *practical skills*. Theoretical knowledge was defined as knowledge that can be found in textbooks or scientific literature. Patients and therapists considered knowledge about anatomy, physiology and pathology as essential components of professional expertise. In addition, both stakeholder groups stressed the importance of pathology-specific exercise therapy.

I like to have information about how I can improve my condition and which specific exercises I need to do. (Patient 3)

The program should be well structured. It should have an adequate selection of exercises. The therapist should reflect on how to dose the exercise. How many repetitions? What are the patient’s goals? What effect do I want to achieve? (Exercise therapist 5)

The practical skills subcategory was defined as abilities that can best be learned by practical experience. The therapist’s capacity to provide the patient with a clear exercise demonstration was important to both stakeholder groups.

I expect the therapist to be able to demonstrate the exercises. That she not only gives a theoretical explanation, but also participates in the exercises shown. (Patient 5)

When the patient is more of a visual learner, the therapist should demonstrate the exercise (Exercise therapist 1)

### Didactic-methodological competence

The main category *didactic-methodological competence* referred to the selection of course topics and methods of instruction. Four subcategories were identified: *topic selection*, *exercise selection*, *teaching method* and *volitional strategies*. The subcategory topic selection described aspects of selecting and structuring the GEP content. Therapists reported that they tried to consider the patients' wishes, expectations and needs when selecting course topics. A coherent structure and sequencing of session topics was identified as a core component from the therapist’s perspective.

Structure and planning are important insofar as they ensure professionalism. A therapist who changes his mind about what he would like to do five times during the course of the GEP session, and then runs out of the room to fetch different training equipment five times because of that, does not seem very competent to me. (Exercise therapist 1)

Topic variety was mentioned by both stakeholder groups as a way of creating a pleasant learning environment and providing patients with different therapeutic approaches.

I think it is brilliant that we covered so many different topics. Sometimes we performed the exercises standing up, then sitting down, lying down or with different training equipment. (Patient 3) Exercises should not be focused just on strength training. I always like to incorporate topics like mobilization, stretching and myofascial release. (Exercise therapist 4)

The subcategory exercise selection was defined as the therapist’s choice of basic and alternative exercises. Patients and therapists referred to the ability to modify an exercise for different situations as important. In particular, patients appreciated the therapist not urging GEP participants into performing an exercise in exactly one way (all or nothing), but that the heterogeneous nature of the group was taken into account. Therapists stated three pre-defined difficulty levels per exercise as being sufficient when trying to account for the disparity in participant performance.

He always gave me something to do. Moreover, I could even do the exercise at the highest difficulty level. That was great! (Patient 1)

It is important to be able to provide different exercise variations. I personally like to show exercises in three difficulty levels. First, I show the basic exercise. Then, when the patient cannot handle that, I show him the easier variant. If it is too easy, I show him the difficult variant. That way, you can cover most group participants. (Exercise therapist 4)

The subcategory teaching-method was defined as the therapist’s ability to explain exercises and apply patient-specific methods of instruction. In this regard, therapists reported that language barriers and comprehension difficulties could be overcome through exercise demonstration, by breaking down tasks into separate steps or by using tactile cues. Volitional strategies for the implementation of unsupervised training and physical activity into the daily life home setting were mentioned by both stakeholders. Whereas patients referred to individualized exercise handouts as being helpful and a source of motivation, therapists emphasized the importance of teaching exercises using affordable and readily available training equipment.

She was able to motivate me by giving me exercise handouts. Something to read up on in case I do not remember the exercise. (Patient 5)

I like show exercises that the patients can easily do at home. For example, I use resistance bands or balance pads. (Exercise therapist 5)

### Personal competence

The main category *personal competence* included statements relating to the therapist’s personal characteristics. The five subcategories deduced were *empathy*, *friendliness*, *humor*, *affinity for sports and exercise*, and *authenticity*. Empathy was important to both stakeholder groups and included sympathy for the patient’s life situation as well as receptiveness to fears and worries expressed by group participants. Whereas friendliness was exclusively referred to by patients, both stakeholders regarded humor as essential.

I expect the therapist to be relaxed and to have a sense of humor. (Patient 3)

I think it is important that there is a certain relaxing atmosphere. That you can laugh together. Because experience has shown me that it is more pleasant this way in group-based exercise therapy. (Exercise therapist 4)

The subcategory affinity for sports and exercise was defined as the therapist’s ability to express a positive attitude towards physical activity. Therapists stressed the importance of leading by example by making their exercise demonstrations mirror their verbal instructions. To this end, a certain level of physical fitness and personal experience with the exercises taught were mentioned as necessary. In contrast, both stakeholder groups also emphasized the value of authenticity, which was defined as showing vulnerability, putting pretenses aside and being oneself. In this regard, authenticity was seen as a way of connecting with patients and sharing feelings.

The therapist should be able to admit when she herself is suffering from back pain. It is important to me, that she is genuine and authentic. (Patient 1)

Yes, I am getting older now, too. I can no longer do every exercise that we teach the patients. So sometimes, I simply tell them: ‘Listen, I too have knee and shoulder problems, which is why I cannot do the difficult exercises. However, you can also benefit from the easier exercises if you do them regularly.’ (Exercise therapist 2)

### Social-communicative competence

The main category *social-communicative competence* comprised therapist-patient communication and interaction. Five subcategories were identified: *motivation*, *patient-centered care*, *group facilitation*, *feedback*, and *maintaining professional boundaries*. Besides verbal encouragement, a motivational strategy stated by both stakeholder groups was the implementation of objective performance tests to monitor and highlight patient progress.

Today, for example, we repeated a performance test that we had already done last week. We had to write down how many seconds we were able to do these exercises. Then we were asked by the therapist to practice the exercises in our spare time. Today we checked again to see if we had improved. I think that if you set these small goals with your patients, you actually motivate them. (Patient 3)

The performance test takes place twice over the course of the program. Besides asking individual patients for their result, I also chart everyone’s result on a whiteboard. That way I can easily visualize potential increases in performance and patients are motivated by their individual progress. (Exercise therapist 5)

Another motivating factor stated by therapists was the promotion of group cohesion by creating a pleasant learning environment conductive to the formation of bonds between group members.

It is important that the therapist manages to create a state of group identity. (Patient 1)

It is incredibly motivating when patients exchange information with each other. The more they communicate, the more they spend time together and are motivated during GEP. This is particularly true for the inpatient rehabilitation setting. The patients have lunch together. Some of them arrange to meet in the evenings. That can create very nice dynamics. (Exercise therapist 1)

The subcategory patient-centered care was defined as the therapist’s ability to respond to the needs of individual participants. To this end, a key aspect mentioned by patients and therapists was the consideration of fears and uncertainties expressed by group participants. Commonly stemming from insufficient knowledge about their respective pathology, patients welcomed the therapist’s professional opinion and reassurance.

It is not that I cannot do the exercises. I simply fear to get hurt again. However, when I have confidence in the therapist and she assures me: ‘You can do this!’ Then I dare to try the exercises. (Patient 1)

Some doctors still advise their patients to rest their back. In that case, I first have to educate and reassure the patient. For example, I have to tell him that in order to stay healthy, intervertebral discs rely on physical activity. (Exercise therapist 4)

To avoid the risk of overlooking quiet and inconspicuous patients, therapists highlighted the importance of dividing their attention as equally as possible between group participants. In addition, memorizing and addressing patients by their family name was described by patients as going the extra mile.

She also addressed people by name, even though we were a group. She really made the effort. (Patient 5)

The subcategory group facilitation was defined as the therapist’s ability to lead group discussions and interactions while remaining impartial. Therapists stressed the long-term value of active, group-based learning instead of front-of-class teaching.

Patients should exchange ideas with each other. That way, they have the opportunity of finding solutions on their own. I like to let them work on everyday problems in the group, because I do not always want to tell them the solution beforehand. Thus, the things they work out do not just go in one ear and out the other. (Exercise therapist 2)

The subcategory feedback was defined as the therapist’s ability to provide patients with information about their performance as well as asking for their opinion regarding course topics, exercise selection and therapist behavior. Although therapist feedback varied in timing and occasion, both stakeholder groups highlighted the importance of giving instructions on movement correction and commending patients for their performance.

She should check if I'm doing the exercises the right way. (Patient 2)

Yes, you should always try to commend patients on their performance. For example, I think it is very important to say: ‘Great Job! You've done this very well!’ (Exercise therapist 2)

Patients and therapists further mentioned the value of collecting and incorporating patient feedback in the planning and structuring of topics and exercises.

It is important to check whether the group is satisfied and what they would like to cover in the next course unit. (Patient 3)

The therapist should frequently ask patients for their feedback. For example: ‘Are you guys missing something? Have you thought of anything that we need to add?’ (Exercise therapist 1)

The subcategory professional boundaries was defined as the therapist’s ability to strike a balance between becoming a trusted confidant to patients and maintaining a sense of professionalism. Both stakeholder groups stated boundaries as beneficial for patient-therapist interaction and maintaining therapist authority.

She did it very well. She was approachable. However, in a professional way, she also clearly emphasized her role as therapist. (Patient 1)

I think it is important for patients to have fun, and I tend to let them have their fun with me, too. However, they also have to know when it is enough. I believe that this is important, especially in closed groups. They have to know who is in charge. (Exercise therapist 3)

## Discussion

This qualitative study aimed to develop an understanding of relevant exercise therapist competencies in the context of GEP. Our findings revealed four competency categories—professional expertise, didactic-methodological competence, personal competence, and social-communicative competence. Furthermore, the spectrum of 16 discrete competency subcategories and respective characteristics indicated that exercise therapists working in GEP were expected to possess a broad range of skills and abilities.

With regard to professional expertise, both groups considered biomedical knowledge and practical therapy skills as essential therapist attributes. Due to the high standards of professional job qualification in Germany, these attributes are routinely taught as part of vocational therapist education [35, 36], and should therefore be mastered by every certified physical therapist and sports scientists. However, exercise therapy in Germany is not limited to biomedical effects, but constitutes a multidimensional treatment [16, 17], including psychosocial, behavioral and educational goals [9]. Consequently, in order to best utilize biomedical knowledge in the realization of biopsychosocial treatments, exercise therapists need to apply a much broader skill set.

Similar to the field of group-based psychotherapy, our results highlight the value of didactic, personal and social-communicative competencies for the implementation of psychosocial effects in GEP. For example, Yalom [14] identified key therapeutic factors and corresponding therapist skills necessary for successful group-based psychotherapy interventions. A subset of Yalom’s therapeutic factors have since been identified in exercise-based treatments like concentrative movement therapy [37] and psychomotor education [15]. Based on our findings regarding relevant exercise therapist competencies, the four therapeutic factors: ‘*instillation of hope*’, ‘*universality*’, ‘*imparting information*’ and ‘*group cohesion*’ are likely to take effect in GEP [14].

In order to reinforce the patients' belief in the effectiveness of the program (‘*instillation of hope’*), therapists can address the fears und uncertainties of group participants through education and reassurance. In addition, verbal encouragement can act as a powerful motivational technique [38, 39] and therapists should aim to address even less talkative group participants [19]. Our data further show that hope and motivation can be increased through repeated and objective performance tests. As a crucial step of the rehabilitation goal-setting process [40, 41], a minimum of two assessments over the course of GEP can highlight patient progress and boost the group’s confidence in the treatment. Since the patients in our study strongly welcomed topic variety, exercise therapists could increase and highlight the effectiveness of GEP by covering a multitude of different therapeutic approaches.

To counter feelings of isolation and aid the therapeutic process, therapists should help their patients realize that others with similar disabilities are participating in the group program (‘*universality*’) [14]. We found that exercise therapists valued patient involvement in the selection and structuring of course topics, thereby offering opportunities for patient interaction and ‘*universality*’. Therapists further stressed the importance of group discussions and independent, goal-oriented problem solving. This indicates that patient interaction in GEP should not be limited to the sharing of emotions [42], but instead contain information to facilitate self-efficacy expectations [43, 44].

Since GEP in Germany can include up to twelve patients with individual goals and medical conditions, exercise therapists face the challenge of adapting to the needs of individual group participants while at the same time covering an extensive course content. Consequently, patient education and instruction strongly depend on the therapist’s didactic and methodological abilities (‘*imparting information*’). Regarding exercise selection and modification, patients seem to value pathology-specific exercises as well as suitable ways of exercise regression and progression. Exercise therapists can meet these expectations by using a pre-defined exercise progression continuum, accounting for different levels of performance and disability [45]. Additionally, when facing comprehension difficulties or language barriers, therapists should apply tactile cueing and stepwise exercise demonstration, both of which represent simple but effective ways of client-centered exercise prescription [46]. With regard to the transition from GEP to a home-based exercise program, therapists ought to focus on teaching exercises using affordable and readily available training equipment. Thus, barriers to exercise adherence such as time-consuming equipment preparation and difficulty in initiating exercises can be avoided [47].

*‘Group cohesion’* can be defined as a dynamic process that reflects the tendency for group participants to remain united in the pursuit of shared goals and/or for the satisfaction of the participants’ needs [48]. It has further been described as the counterpart to the patient-therapist relationship in individual therapy [14] as well as a basic feature of group-based exercise programs [45]. Concerning the effects of group cohesion in exercise therapy, meta-analytic summaries indicate that exercising in groups is associated with positive attitudes and cognitions towards physical activity, including self-efficacy expectations [49]. Regrettably, besides mentioning the value of a pleasant learning environment and relaxing atmosphere, the therapists in our study did not elaborate on how to best promote group cohesion in the context of GEP. Since team-building strategies like partner work, collective goal setting and problem solving can promote cohesion in group-based exercise classes [49] their use in GEP seems warranted.

Finally, our findings regarding the importance of empathy, friendliness and authenticity are supported by data from the fields of psychotherapy [50, 51] and group-based rehabilitation [19]. In particular, empathy and authenticity were highlighted by Yalom [14] as the basis of the patient-therapist relationship and prerequisites of every therapeutic factor. However, our data also attest to the importance of professional boundaries, which are intended to define a safe interaction between exercise therapists and their patients. Recommendations on how to establish and maintain professional boundaries in the context of physical and occupational therapy have previously been published by several professional associations [52–54]. Consequently, it is advisable that exercise therapists balance their use of empathy and authenticity, lest they undermine their credibility and endanger the professional patient-therapist relationship.

### Strengths and limitations

The results of our study provide a novel insight into the multitude of relevant therapist competencies in GEP. Besides professional expertise, key didactic, personal and social-communicative skills needed for the psychosocial effects of exercise therapy have been highlighted. Despite these findings, there are also limitations to this study. First, due to the monocentric design, the recruited participants may not have been representative of patients and exercise therapists in other inpatient rehabilitation clinics. In addition, the self-selection of the interviewees (convenience sampling) might limit external validity. Furthermore, the specific content and conceptual framework of the clinic’s GEP program might have influenced our findings. For example, only two patients had previously taken part in other rehabilitation programs and most patients based their answers on their experience with the clinic’s GEP. There is also the possibility of other key therapist competencies not specifically mentioned by the study’s participants. For instance, some competencies might have been taken for granted or gone unnoticed since the program’s exercise therapist did not stand out negatively in these regards. Finally, the sample in this study was small, which limits the generalizability of our findings.

### Clinical implications and outlook

Our data highlight the importance of didactic, personal and socio-communicative therapist competencies in the context GEP. While only insufficiently covered by competency frameworks of the respective national governing bodies for sports and physical therapists [21–24], more research is needed to review the extent to which these skills are addressed in the current vocational and continuing education of exercise therapists. In addition, future studies should aim to check our results with larger sample sizes and in the context of other inpatient rehabilitation facilities. Since our data only provided limited information on how to promote group cohesion in the context of GEP, a more in-depth exploration of promoting factors is necessary.

Although the use of therapist feedback as a didactic and motivational tool is supported by a large body of literature [55–57], our data showed a strong variation in content, occasion and timing of feedback use in GEP. This suggests an intuitive rather than structured application of feedback by exercise therapists. Consequently, further research should aim to identify the optimal implementation of feedback in GEP.

## Conclusion

The present study shows that exercise therapists working in the context of group-based exercise programs are expected to possess a broad range of competencies. In particular, didactic-methodological, personal as well as social-communicative abilities are needed in order to realize the biopsychosocial determinants of exercise therapy and achieve the respective rehabilitation goals. Further research is needed to determine whether these abilities are sufficiently covered in the respective vocational and continuing education programs.

## Data Availability

The datasets used and/or analyzed during the current study are available from the corresponding author on reasonable request.

## Abbreviations

COREQ: Consolidated criteria for reporting qualitative studies
DRKS: German Clinical Trial Register
GEP: Group-based exercise programs
SD: Standard deviation
